# Phenotypic divergence in sleep and circadian cycles linked by affective state and environmental risk related to psychosis

**DOI:** 10.1093/sleep/zsac311

**Published:** 2022-12-14

**Authors:** Ross J Purple, Jan Cosgrave, Iona Alexander, Benita Middleton, Russell G Foster, Kate Porcheret, Katharina Wulff

**Affiliations:** School of Physiology Pharmacology and Neuroscience, University of Bristol, Bristol, UK; Sleep and Circadian Neuroscience Institute, Nuffield Department of Clinical Neurosciences, Sir William Dunn School of Pathology, University of Oxford, Oxford, UK; Sleep and Circadian Neuroscience Institute, Nuffield Department of Clinical Neurosciences, Sir William Dunn School of Pathology, University of Oxford, Oxford, UK; Department of Chronobiology, Faculty of Health and Medical Sciences, University of Surrey, Guildford, UK; Sleep and Circadian Neuroscience Institute, Nuffield Department of Clinical Neurosciences, Sir William Dunn School of Pathology, University of Oxford, Oxford, UK; Norwegian Centre for Violence and Traumatic Stress Studies, University of Oslo, Oslo, Norway; Institute of Clinical Medicine, Faculty of Medicine, University of Oslo, Oslo, Norway; Department of Radiation Sciences and Department of Molecular Biology, Umeå University, Umeå, Sweden; Wallenberg Centre for Molecular Medicine (WCMM), Umeå University, Umeå, Sweden

**Keywords:** environmental risk factors, psychosis, sleep EEG, actigraphy, melatonin

## Abstract

**Study Objectives:**

Environmental cues influence circadian rhythm timing and neurochemicals involved in the regulation of affective behavior. How this interplay makes them a probable nonspecific risk factor for psychosis is unclear. We aimed to identify the relationship between environmental risk for psychosis and circadian timing phenotypes sampled from the general population.

**Methods:**

Using an online survey, we devised a cumulative risk exposure score for each of the 1898 survey respondents based on 23 empirically verified transdiagnostic risks for psychosis, three dimensions of affect severity, psychotic-like experiences, and help-seeking behavior. Quantitative phenotyping of sleep and circadian rhythms was undertaken using at-home polysomnography, melatonin and cortisol profiles, and 3-week rest–activity behavior in individuals with a high-risk exposure load (top 15% of survey respondents, *n* = 22) and low-risk exposure load (bottom 15% of respondents, *n* = 22).

**Results:**

Psychiatric symptoms were present in 100% of the high-load participants and 14% of the low-load participants. Compared to those with a low-load, high-load participants showed a later melatonin phase which was reflected by a greater degree of dispersion in circadian timing. Phase relationships between later circadian melatonin phase and later actigraphic sleep onsets were maintained and these were strongly correlated with self-reported sleep mid-points. No differences were identified from polysomnography during sleep between groups.

**Conclusion:**

Distinguishing circadian timing from other sleep phenotypes will allow adaptation for dosage of time-directed intervention, useful in stabilizing circadian timekeeping physiology and potentially reducing the multisystemic disruption in mental health disorders.

## Graphical Abstract

**Graphical Abstract:**
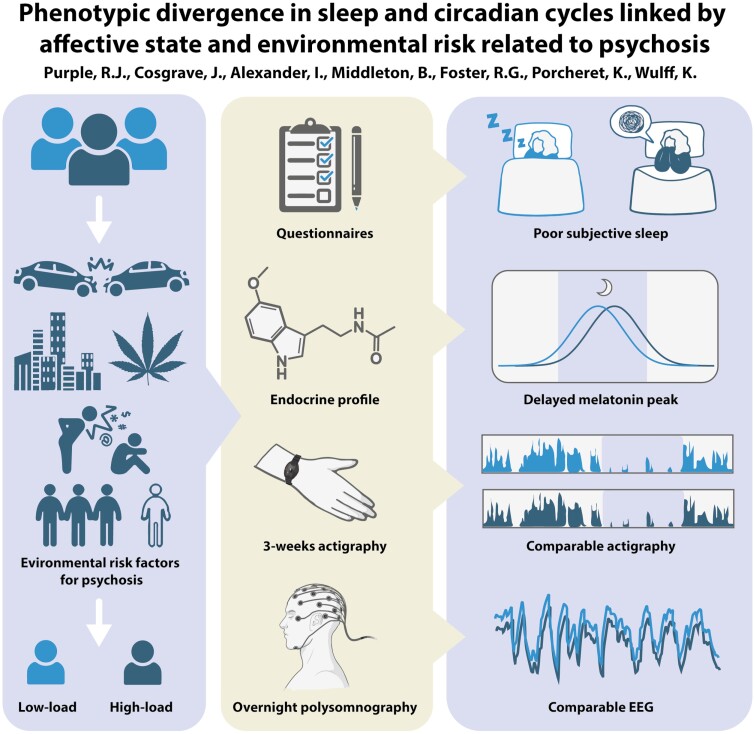

**Statement of Significance**

We show that participants with a high load of environmental risk for psychosis have a delayed circadian profile of melatonin compared to controls. This suggests the delayed circadian phase that we have previously found in patients with schizophrenia is also present in individuals with a “high-risk” profile but not seeking medical help, hence will not be on the radar of clinical professionals. This study highlights the need to distinguish circadian timing from other sleep phenotypes with intervention potentially modeling personalized light therapy for circadian phase realignment, thereby reducing the development of multisystemic disruption in mental health.

## Introduction

Our circadian timekeeping system is an important driver for regulatory hierarchies of brain and body functions that allows nervous, endocrine, and metabolic processes to alternate their activity according to daily and seasonal changes [1, 2]. This dynamic ensemble contributes to an individual’s overall circadian phenotype, evolved to match phases of physical, emotional, and cognitive performance to the appropriate time of day. Light exposure, physical activity, and food intake are among the strongest temporal external variables to which the circadian timekeeping system is sensitive to and responds with synchronization. Regular exposure to sufficient light during the daytime ensures a stable phase synchronization of internal rhythms with the geophysical environment. However, irregular exposure lowers the strength of this synchronization, and it depends instead on individual behavior, i.e. routines/no routines, bedtime, get-up time, excessive screen time, electrical indoor light or daylight, mealtimes or snacking, social life, or isolation [3].

Psychosis is present on a spectrum of severity within the general population, including psychotic-like experiences, psychotic symptoms, and psychotic disorders such as schizophrenia [4]. Environmental (as well as genetic) risks for psychosis are often nonspecific, meaning that they are also carriers of risk for the development of other mental health disorders. High rates of affective symptoms in the development of psychosis have been highlighted in general population samples, suggesting that these risks are partly mediated by pathways of affective dysregulation [5]. During development, the human brain is subject to modification via environment and experience for which it requires qualitatively different solutions to mature [6, 7]. A history of birth complications or childhood adversity represents replicated, transdiagnostic environmental risk exposures for the developing brain, a possible trajectory to develop a vulnerability to later affective suffering [5, 7, 8].

Sleep and circadian timing are known to interact with both affective state and psychosis symptoms [9]. We have previously shown a large heterogeneity in sleep and circadian rhythms in individuals diagnosed with schizophrenia [10] and others have shown that circadian disturbance can be predictive of psychotic symptom severity [11]. A longitudinal actigraphic study in community-dwelling individuals with schizophrenia found an increase in self-reported severity of both psychotic symptoms and negative affect followed by shorter night sleep periods [12]. Sleep efficiency was only associated with psychosis severity on days with high levels of negative affect [12]. In another study, morning negative affect partially mediated the association between night sleep fragmentation and next-day impaired functioning and psychotic symptom severity in individuals with schizophrenia [13]. In individuals with an ultra-high risk (UHR) for psychosis, Shetty et al. documented subjective sleep disturbance to be associated with depressive and attenuated positive psychotic symptoms, yet, evening preference was specifically associated with negative symptomatology [14]. On this note, altered circadian timing, which has previously been identified to result from exposure to early-life adversities, is suggested to have a mediating role in the development of affective dysregulation [7]. We further speculate that affective dysregulation may also become a mediator on subsequent relationships between circadian lateness and psychosis outcome. In this light, the tight relationships between environmental risk exposure, sleep and circadian timing, affective dysregulation, and psychosis may reflect dynamic cyclical interactions rather than linear chronological causality [15]. However, collectively assessing how these factors co-occur within the same individuals remains to be explored.

For this study, we recruited from an online survey (OWLS [8]) assessing cumulative environmental risk exposure for psychosis and recorded high temporal resolution physiological circadian and sleep parameters. Instead of restricting to clinical phenomenology, such as when assessing UHR individuals, a cumulative risk factor approach was employed since this adds to a more comprehensive picture through which aggregated risk from many individual risk factors can be assessed. Our research question was: Do individuals with a high load of environmental risk exposure for psychosis differ in their sleep and circadian timing from those, who experience less exposure to environmental risks? We aimed to test, whether sleep and circadian profiles differ as a function of exposure to environmental risks. We focused on (1) characterizing, evaluating, and defining the roles of circadian rhythms and sleep profiles with respect to existing psychological phenotypes in these groups of individuals, and (2) examining the relationship between objective physiological profiles against subjective self-reported assessments. Based on our previous findings in schizophrenia patients [10], we hypothesized that higher levels of environmental risk exposures would be paralleled by poorer subjective sleep and greater heterogeneity in circadian timing, especially hypothesizing a late circadian phenotype. After testing for these associations, we assessed by exploratory post hoc analyses, whether late circadian phenotypes were associated with more severe sleep and mental state symptoms among individuals with higher environmental risk exposures.

## Methods

### Study participants

Participants were recruited from an online (Oxford Wellbeing, Life and Sleep [OWLS]) survey [8] assessing established risk factors for psychosis, psychotic-like experiences, symptoms of depression, anxiety and stress, and help-seeking behavior for 1898 individuals of the general population (see Supplementary Methods for further details). “High-load” risk exposure participants (High-load) were selected from the top 15% of individuals in this survey for risk factors and symptoms endorsed, with “Low-load” risk exposure (Low-load) participants selected from the bottom 15%. Participants were aged 18–30 years and were excluded if they had been working night shifts in the last 3 months, traveled across more than one time zone in the past 2 weeks, were pregnant, had epilepsy, or had taken any medication for a mental health problem (including sleep problems) within the last 3 months. In addition, Low-load exposure participants were excluded if they had a history of any mental health disorder and high-load exposure participants were excluded if they had a history of a diagnosed psychotic disorder or psychotic episode. All participants provided written informed consent and received an honorarium for their time. The study was conducted in accordance with the Declaration of Helsinki and approved by the North-West Liverpool Central NHS Research Ethics Committee (REC: 14/NW/1142).

### Experimental design

During the 21-day protocol, participants completed questionnaires/interviews on their mental state, personality, and sleep, and underwent a 48-h urinary melatonin assessment, 2 nights of domiciliary polysomnography (PSG), 2 mornings of salivary cortisol collection, and 3 weeks of actigraphy. Participants also completed further tasks during the second night of PSG to measure sleep-related memory consolidation, which is published separately [16].

### Mental state assessments

The Mini International Neuropsychological Interview (MINI) was used to screen for major Axis I psychiatric disorders in DSM-IV and ICD-10 [17] and the Comprehensive Assessment of At-Risk Mental States (CAARMS) to assess clinical risk (ultra-high-risk status) for positive symptoms of psychosis with or without a drop in social functioning [18]. Participants were assessed on a case-by-case basis and borderline cases were discussed with the research team and a clinical psychologist.

### Questionnaires

The Pittsburgh Sleep Quality Index (PSQI) [19] was used to assess subjective sleep quality with a global score of more than 5 being indicative of poor sleep quality and component scores assessing subjective sleep quality, sleep onset latency, sleep duration, sleep efficiency, and daytime dysfunction. The short version of the Sleep Condition Indicator (SCI-2) [20] was used to determine symptoms of insomnia with a score below 4 indicating diagnostic criteria for insomnia disorder. The SLEEP-50 [21], IOWA Sleep Experiences Survey (ISES [22]), Nightmare Frequency Questionnaire (NFQ [23]), Nightmare Distress Questionnaire (NDQ [24]), and Munich Chronotype Questionnaire (MCTQ [25, 26]) were used to assess sleep disorders, unusual sleep experiences, frequency and daytime impact of nightmares, and chronotype and social jetlag, respectively. Manic symptomology, neuroticism, schizotypy, trait dissociative symptoms, absent-mindedness, and trait mind wandering/day-dreaming behavior were assessed using the Mood Disorder Questionnaire (MDQ [27]), neuroticism subscale of the Eysenck Personality Questionnaire (EPQ-N [28]), Schizotypal Personality Questionnaire Brief Revised (SPQ-BR [29]), Dissociative Experiences Scale (DES [30]), Cognitive Failures Questionnaire (CFQ [31]), and Mind Wandering Questionnaire (MWQ [32]).

### Circadian rhythm assessments

A 48-h profile of 6-sulphatoxymelatonin (aMT6s, a metabolite of melatonin) was taken to estimate the circadian period and phase of the participant’s circadian pacemaker. Participants were asked to record the volume and time of each passing of urine using a bottle provided, and aliquot two 5-mL samples of each collection. A radioimmunoassay of melatonin sulfate using AN^123^ was performed by Stockgrand Ltd (University of Surrey) [33].

To determine circadian rhythm status in melatonin sulfate, a nonlinear least square method was applied to account for unequally spaced collection times: *MT = c +* (*Amp* × cos(2*π* × ((*t−tc*)/*T*))) where *MT* = aMT6s secretion rate (ng/h) and *t* = time. The parameter estimates are *c* = mesor (rhythm-adjusted mean), *Amp* = amplitude, *tc* = phase angle (time of aMT6s concentration peak or acrophase relative to a reference point in time) and *T* = the period. Nonlinear regression was performed using SAS software for Windows, version 8. Data from nine individuals were excluded from this analysis because of nonsignificant fits due to insufficient sampling or low amplitude. Inaccurate data from four individuals were inspected and corrected in data collection (see Supplementary Methods).

### Actigraphy

Participants wore an actiwatch with an integrated light sensor (MotionWatch 8; CamNtech Ltd, Cambridge, UK) on their nondominant wrist and kept a standardized diary of daily bedtimes, get-up times, and daytime activities. Actigraphy data were sampled in 1-min epochs and analyzed with version 1.1.15 of the CamNtech MotionWare software (CamNtech Ltd.). Rest–activity patterns were annotated using diary data and a “bedtime” and “get-up time” were manually entered with “sleep start” and “sleep end” calculated automatically using medium sensitivity (threshold level of 40 counts) to correspond with the settings used in the validation by PSG [34]. Average sleep parameters including Sleep Onset, Sleep Offset, Total Sleep Time (TST), Wake After Sleep Onset (WASO), Fragmentation Index (FI), and Sleep Efficiency (SE) (defined as the ratio of TST to the Sleep Period [i.e. time from automatically determined Sleep Onset to Sleep Offset, including WASO and excluding sleep latency]) were calculated by averaging across days whereas variability in sleep parameters was calculated by taking the standard deviation across days. A Non-Parametric Circadian Rhythm Analysis (NPCRA) was also performed using the MotionWare software to detect amplitudes for the highest 10-h activity (M10 counts) and lowest 5-h activity bins (L5 counts) from a moving average across the time series with a 1-h window, which was used to derive the Relative Amplitude (RA: 0 = Gaussian noise to 1 = perfect rhythm), Interdaily Stability (IS: 0 = Gaussian noise to 1 = perfect stability) and Intradaily Variability (IV: 0 = perfect sine wave to 2 = Gaussian noise). One participant was removed from actigraphy analysis due to insufficient wearing of the actiwatch. Light exposure intensity and timing concurrently recorded with each actigraphic device have not yet been analyzed as it comprises a richly layered investigation on its own. See Supplementary Methods for further information on actigraphic variables calculated.

### Polysomnography

Domiciliary polysomnography (PSG) was recorded with the SomnoScreen+PSG from SOMNOmedics GmbH using a standard bilateral montage of 11 channels (Fp1, Fp2, F3, F4, C3, Cz, C4, P3, P4, O1, and O2) referenced to the mastoids. Signals were sampled at 128 Hz and digitally filtered using finite impulse response band-pass filtering between 0.2 and 35 Hz with a Hamming window function applied. All PSG was set up in the laboratory and data were collected in the participant’s home environment where they were encouraged to follow their usual daily routines. The first night acted as an adaptation night, and the data were analyzed from the second night’s recording [35]. PSG data were analyzed using the DOMINO version 2.6.0 software (SOMNOmedics GMbH) and scored at 30 s epochs according to the American Academy of Sleep Medicine criteria with the separation of both stage 3 and stage 4 sleep to gain greater macrostructural detail [36]. Spectral and sleep spindle analysis was performed using MATLAB (The MathWorks Inc, Natick, MA). Power density was calculated using a fast Fourier transform (FFT) with a 5-s window length, 0% overlap, and a frequency resolution of 0.2 Hz. Artifacts were automatically removed as whole epochs using a low (0.6–4.4 Hz) and high frequency (20–40 Hz) moving median filter with a 10-s window length. For NREM sleep, an average of 84.42% ± 8.63% of epochs were kept after artifact removal. To compare power densities, the FFT data were binned into delta (0.6–4 Hz), theta (4.2–8 Hz), alpha (8.2–12 Hz), sigma (12.2–16 Hz), and beta (16.2–20 Hz) frequency bands. To detect sleep spindles, an automatic detection algorithm was used based on previous methodologies [37] and limited to artifact-free NREM sleep. Both spindle detection and spectral analysis were carried out on electrodes Fp2, F4, C4, P4, and O2, but results in the main text are consistently shown from channel C4 only. See Supplementary Methods for further information on spindle detection.

### Cortisol awakening response

As a measure of HPA axis function, the cortisol awakening response (CAR) was calculated from salivary cortisol levels [38]. Sampling was carried out at four time points (immediately after waking then 15, 30, and 45 min after waking) on two mornings at home. Participants were asked to maintain low activity levels, not to eat, drink, brush their teeth, smoke after waking up, and not consume alcohol the night before. A radioimmunoassay using ^125^I cortisol was performed by Stockgrand Ltd (University of Surrey). To calculate the cortisol awakening response, the area under the curve with respect to increase (AUC_i_) was used since it emphasizes changes over time and sensitivity of the response [39, 40]. Data from one participant were excluded due to a failure to detect cortisol.

### Statistical analysis

All statistical analysis was computed using “R” software [41]. Group differences were calculated using ANOVA, post hoc *t*-tests for normal data, nonparametric Wilcoxon rank-sum tests for non-normal data, and Chi-squared tests for count data. For cortisol awakening response, a repeated-measures analysis of variance was used to compare cortisol levels across the four time points. A Bonferroni correction was applied to α-values to control for multiple testing, where appropriate.

## Results

### Demographics and mental state

Forty-four individuals (2.32% of the entire OWLS sample) completed the study. On average, the High-load group reported 6.8 ± 1.5 risk factors compared to 2.1 ± 0.9 in the Low-load group. A traumatic experience, being born during winter or spring, and having a first-degree family history of a psychiatric disorder were amongst the most prevalent risk factors identified in the entire sample (Supplementary Figure S1). At group level, High-load exposure individuals scored higher on all assessments of mental state and personality (Table 1).

**Table 1.** Demographics of High-load and Low-load groups with risk factors for psychosis

Demographics and mental state	High-load	Low-load	Test-value	*p*
Mean age	22 ± 3	22 ± 3	255.00”	.768
Female	72.73% [16]	68.18% [15]	0.00^	1
Student	59.09% [13]	77.27% [17]	2.72^	.099
Psychotic-like experiences (PQ-16)	9.00 ± 2.62	0.77 ± 1.23	484.00”	<.001
Risk factors for psychosis (mean number)	6.77 ± 1.54	2.09 ± 0.87	484.00”	<.001
Depression (DASS-21)	23.00 ± 10.32	4.01 ± 4.21	470.50”	<.001
Anxiety (DASS-21)	17.64 ± 9.21	2.73 ± 2.51	479.00”	<.001
Stress (DASS-21)	26.18 ± 8.23	4.91 ± 4.26	482.50”	<.001
Help seeking	63.64% [14]	0.00% [0]	17.71^	<.001
FH+, first degree any diagnosis	50.00% [11]	4.55% [1]	9.28^	.002
FH+, 1st degree psychosis	4.55% [1]	0.00% [0]	0.00^	1
Clinical Psychosis Risk (CAARMS)	77.27% [17]	0.00% [0]	24.54^	<.001
With deficit in social functioning	40.91% [9]	0.00% [0]	–	–
Without deficit in social functioning	36.36% [8]	0.00% [0]	–	–
Total mental health items (MINI)	86.36% [19]	13.64% [3]	20.46^	<.001
Depressive disorder	31.82% [7]	0.00% [0]	–	–
Anxiety disorder	72.73% [16]	4.55% [1]	–	–
PTSD	9.09% [2]	0.00% [0]	–	–
OCD	13.64% [3]	0.00% [0]	–	–
Eating disorder	13.64% [3]	0.00% [0]	–	–
Alcohol/substance dependence	31.82% [7]	9.09% [2]	–	–
Suicidality	40.91% [9]	0.00% [0]	–	–
(Hypo) Manic episode	63.64% [14]	0.00% [0]	–	–
Mania (MDQ)	22.73% [5]	0.00% [0]	3.61^	.057
Schizotypy (SPQ-BR)	67.59 ± 12.79	24.36 ± 13.59	479.50”	<.001
Neuroticism (EPQ)	16.41 ± 3.54	4.96 ± 4.73	462.50”	<.001
Dissociation (DES)	13.21 ± 6.61	4.01 ± 3.40	437.00”	<.001
Cognitive failures (CFQ)	53.68 ± 12.76	33.45 ± 12.82	421.00”	<.001
Mind wandering (MWQ)	21.09 ± 4.07	16.36 ± 4.58	372.50”	.002

Shown are means ± standard deviation or % [count]. Symbols denote different tests: “ = Wilcoxon rank sum test, ^ = chi-squared test. FH = family history.

### Circadian profiles

**Figure 1.**
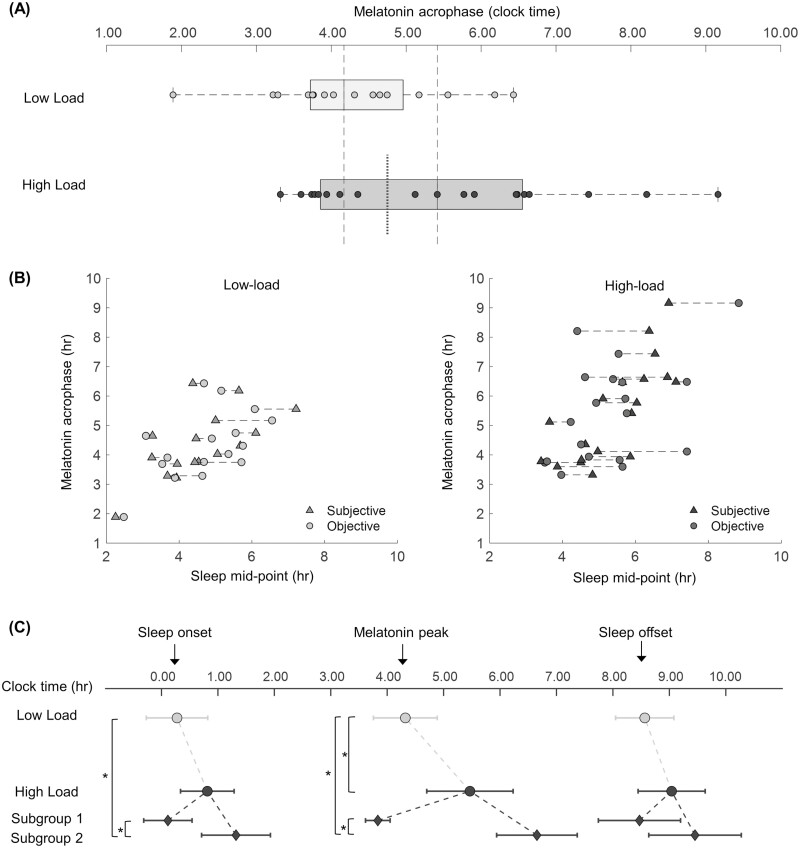
Melatonin metabolite profiles. (A) Boxplots of melatonin peak between groups of high and low loads of risk exposure. Circles represent single individuals. The vertical dashed lines indicate the medians, the left and right edges of the box indicate the 25th and 75th percentiles, and the whiskers extend to the most extreme data points not considered outliers. The vertical dotted line indicates the subgroup split for high-load individuals. *N* = 35. (B) Scatterplots of melatonin peak in relation to subjective (Munich Chronotype Questionnaire, MCTQ) and objective (actigraphic) sleep mid-points with dashed connections highlighting discrepancies between subjective and objective assessments. (C) Relationship between habitual sleep onset and sleep offset times and internal circadian time derived from melatonin metabolite. Whiskers denote 95% confidence intervals of the mean *N* = 35 (High-load: *n* = 19 (subgroup 1: *n* = 8, subgroup 2: *n* = 11), Low-load: *n* = 16). Asterisks denote significance at *p* < .05.

Individuals with high-load exposure showed a significantly later melatonin acrophase by an average of 1.15 h (*t* = 2.36, *p* = .025; Figure 1A, Table 2). Melatonin acrophase was strongly correlated to subjective sleep mid-point (*r* = 0.74, *p* < .001) and moderately correlated to actigraphic sleep mid-point (*r* = 0.53, *p* < .001; Figure 1B). Both groups had similar mean melatonin levels (*t* = 1.32, *p* = .198, Table 2) and melatonin amplitudes (*t* = 1.29, *p* = .209, Table 2). A significantly later subjective sleep mid-point (MCTQ-MSFsc) by an average of 51 min in high-load exposure individuals was not confirmed by objective-actigraphic sleep mid-points (but on average this was still later by 35 min in the High-load group; Table 2). The discrepancy between subjective and objective sleep mid-points with reference to melatonin acrophase occurs in both directions (Figure 1B dashed lines between dots; see Supplementary Figure S2 for group differences within the same plot).

**Table 2.** Parameters of melatonin rhythms, habitual sleep timing (subjective), and rest–activity rhythms (objective) between groups of high-load and low-load risk exposure

Circadian parameters		High-Load	Low-Load	test-value	p-value
Melatonin metabolite (n=35)	Acrophase (hr:min)	05:28 ± 1:42	04:19 ± 1:09	2.36^#^	0.025
	Mean level (ng/hr)	1056.37 ± 645.96	843.88 ± 247.88	1.32^#^	0.198
	Amplitude (ng/hr)	1208.65 ± 849.64	940.36 ± 283.46	1.29^#^	0.209
Habitual Sleep MCTQ (n=44)	Social jetlag (SJL, min)	88 ± 54	68 ± 50	287.50”	0.290
	Sleep mid-point (MSFsc, hr:min)	05:28 ± 1:09	04:37 ± 1:10	339.50”	0.023
Actigraphy (n=43)	Pseudo social jetlag (SJL, min)	30 ± 44	34 ± 45	-0.30^#^	0.765
	Sleep mid-point (hr:min)	05:19 ± 1:17	04:44 ± 1:15	1.54^#^	0.132
	Sleep onset (hr:min)	00:52 ± 1:05	00:24 ± 1:10	1.39^#^	0.172
	Sleep offset (hr:min)	09:05 ± 1:14	08:33 ± 1:19	1.37^#^	0.177
	Level of activity (M10 counts)	16096 ± 3103	16933 ± 4507	-0.71^#^	0.485
	Level of inactivity (L5 counts)	1083 ± 472	1027 ± 463	0.39^#^	0.696
	Interdaily stability (IS)	0.39 ± 0.10	0.36 ± 0.07	1.01^#^	0.317
	Intradaily variability (IV)	0.94 ± 0.22	1.00 ± 0.19	-1.03^#^	0.308
	Relative amplitude (RA)	0.876 ± 0.043	0.880 ± 0.055	-0.27^#^	0.788

Shown are means ± standard deviation. Symbols denote different tests: ^#^= t-test, “= Wilcoxon rank-sum test.

Since a high degree of heterogeneity in melatonin acrophase was identified in the High-load group, a post hoc exploratory analysis was performed to explore the relationship between melatonin peak and other measures of sleep and mental state. For this, High-load individuals were split into two subgroups based on the distribution of melatonin peaks across individuals (earlier or later than 0445 h; Figure 1A, dotted line). The melatonin peaks of the High-load 1 subgroup (*n* = 8) were within the range of Low-load exposure individuals (ranging from 0332 h to 0435 h). In contrast, the melatonin peaks of the High-load 2 subgroup (*n* = 11) had later melatonin peaks compared to both, the High-load 1 and Low-load individuals (ranging from 0512 to 0916 h, Figure 1C, ANOVA: *F* = 21.85, *p* < .001). This difference was further highlighted through subjective sleep mid-points, which were approximately 1.5 h later in the High-load 2 subgroup compared to both the Low-load and High-load 1 groups (KW test: *X*^2^ = 10.81, *p* = .005). No significant differences were identified in objective sleep mid-points based on actigraphy (*F* = 1.99, *p* = .154). However, relative to clock time, High-load 2 individuals showed significantly later actigraphic sleep onsets compared with High-load 1 and Low-load individuals (*F* = 4.65, *p* = .017). When using the melatonin peak as a phase reference point of circadian time, their sleep started at an earlier circadian phase. Actigraphic sleep offset timing was slightly later in the High-load 2 subgroup without being statistically significant (*F* = 2.37, *p* = .110). High-load 1 individuals did not significantly differ from those of Low-load individuals in melatonin acrophase, subjective sleep onset, and sleep mid-points. A further assessment of subgroup differences can be found at the end of the results section.

### Subjective and objective sleep

High-load exposure individuals reported significantly poorer subjective sleep quality (PSQI) and greater severity on the insomnia scale (SCI-2). PSQI component scores revealed that High-load exposure individuals reported not only significantly poorer sleep, but also longer sleep onset latencies and greater daytime dysfunction. High-load exposure individuals also experienced significantly more nightmares (NFQ) but were not more distressed by nightmares. Responses to unusual sleep experiences and specific sleep disorders were similar between groups (Table 3). There were also no differences between groups in objective sleep parameters as determined by 3-week actigraphy and overnight home polysomnography. This included a similar total sleep time, sleep efficiency, wake after sleep onset, time spent in each sleep stage, and variability in sleep parameters across days (Table 3). While there is consistency within methods of measurements, there are discrepancies between different methods: TST is very similar between actigraphy and PSG, but self-reported TST differs by nearly 2 h. WASO differs between actigraphy and PSG by nearly 1 h, and actigraphically derived sleep efficiency differs by about 10% from PSG and self-reported sleep.

**Table 3.** Sleep parameters by different methods in high- and low-load exposure groups

Sleep parameters by method		High load	Low load	Test value	*p*
Subjective sleep	Global sleep quality (PSQI)	7.00 ± 2.16	3.40 ± 1.97	428.50”	<.001
	Subjective sleep quality	1.73 ± 0.77	0.59 ± 0.67	411.00”	<.001
	Total sleep time (TST; h)	8.64 ± 0.91	8.69 ± 1.09	228.50”	.76
	Sleep onset latency (SL; min)	44.77 ± 34.96	23.00 ± 26.42	372.00”	.002
	Sleep efficiency (S; %)	95.58 ± 5.01	97.62 ± 3.08	171.00”	.09
	Daytime dysfunction	1.73 ± 0.88	0.64 ± 0.66	398.00”	<.001
	Sleep condition indicator-2 (SCI-2)	3.09 ± 2.20	6.91 ± 1.82	51.00”	.001
	Nightmare frequency (NFQ, per month)	2.99 ± 4.17	0.59 ± 0.75	362.00”	.005
	Nightmare distress (NDS)	7.86 ± 9.59	2.55±4.93	309.00”	.092
	Unusual sleep experiences (IOWA)	1.75 ± 1.78	1.14 ± 1.35	290.00”	.233
	Sleep disorder symptoms (SLEEP-50)	59% [13]	23% [5]	4.61^	*.032*
Actigraphy sleep parameter	Total sleep time (TST; h)	6.86 ± 0.77	6.79±0.63	0.34^#^	.74
	Sleep efficiency (SE; %)	81.76 ± 4.01	81.94±4.84	-0.13^#^	.90
	Wake after sleep onset (WASO; min)	81.04 ± 22.65	82.01 ± 26.03	-0.13^#^	.90
	Fragmentation index (FI)	28.01 ± 5.82	27.86 ± 6.98	0.08^#^	.94
	Sleep variability (St.Dev between days)				
	Total sleep time (TST; h)	1.27 ± 0.44	1.06 ± 0.53	1.38^#^	.18
	Sleep efficiency (SE; %)	4.41 ± 1.41	3.88 ± 1.23	1.32^#^	.19
	Wake after sleep onset (WASO; min)	28.09 ± 10.35	25.77 ± 10.94	0.71^#^	.48
	Fragmentation index (FI)	8.84 ± 1.85	8.18 ± 2.42	1.01^#^	.32
EEG sleep parameter	Total sleep time (TST; h:min)	6:48 ± 1.13	6:33 ± 1:02	0.71^#^	.48
	Sleep onset latency (SOL; min)	18.90 ± 15.73	16.26 ± 18.17	0.52^#^	.61
	REM onset latency (ROL; min)	81.41 ± 38.90	84.39 ± 28.10	−0.29^#^	.77
	Sleep efficiency (SE; %)	90.97 ± 6.53	91.66 ± 4.51	−0.41^#^	.69
	Time in stage 1 (min)	21.20 ± 13.79	26.33 ± 11.57	−1.34^#^	.19
	Time in stage 2 (min)	189.36 ± 44.03	181.66 ± 44.66	0.58^#^	.57
	Time in stage 3 (min)	43.00 ± 16.38	41.23 ± 19.21	0.33^#^	.74
	Time in stage 4 (min)	59.91 ± 29.21	55.61 ± 30.06	0.48^#^	.63
	Time in REM (min)	94.31 ± 28.64	88.50 ± 21.50	0.76^#^	.45
	Wake after sleep onset (WASO; min)	20.43 ± 15.29	19.15 ± 10.34	0.33^#^	.75
	Number of sleep stage changes (/h)	15.67 ± 4.33	18.81 ± 4.37	−2.40^#^	*.02*
Power density	Delta power (0.6–4 Hz; µV^2^)	142.20 ± 31.06	130.30 ± 43.98	1.04^#^	.31
	Theta power (4.2–8 Hz; µV^2^)	10.96 ± 3.34	10.92 ± 4.12	0.04^#^	.97
	Alpha power (8.2–12 Hz; µV^2^)	4.34 ± 2.38	4.50 ± 2.36	−0.21^#^	.83
	Sigma power (12.2–16 Hz; µV^2^)	2.94 ± 1.35	2.94 ± 1.19	−0.00^#^	1.00
	Beta power (16.2–20 Hz; µV^2^)	0.51 ± 0.21	0.49 ± 0.17	0.45^#^	.65
Sleep spindles	Total number	1548 ± 304	1551 ± 295	−0.03^#^	.98
	Density (N/min)	6.49 ± 0.61	6.63 ± 0.53	−0.81^#^	.42
	Duration (s)	0.97 ± 0.04	0.98 ± 0.04	−0.35^#^	.73
	Amplitude (µV)	14.90 ± 3.19	15.05 ± 2.95	−0.16^#^	.88
	Frequency (Hz)	13.17 ± 0.28	12.97 ± 0.26	2.32^#^	.03

Data show means ± standard deviation or % [count] where relevant. Symbols denote different tests: ^#^ = *t*-test, “ = Wilcoxon rank-sum test, ^ = chi-squared test. Italics denote significance at the 0.05 level but not after controlling for multiple testing. Note the PSQI and SCI-2 cutoff scores indicate poor sleep quality is >5 and <4, respectively.

The power density during NREM sleep was similar between groups, although a slight nonsignificant shift in sigma power (11–16 Hz) was identified in the High-load exposure group (Supplementary Figure S3A). Sleep spindle analysis revealed a slightly, yet significant, higher spindle frequency of central and parietal sleep spindles in High-load exposure individuals (C4: *t* = 2.32, *p* = .03; P4: Supplementary Table S3), which matched the identified shift in sigma power. No other differences in spindle characteristics were found between groups (Table 3, Supplementary Figure S3B). A secondary analysis was performed to describe spindle density for High-load subgroups based on clinical psychosis risk (positive screens on the CAARMS with and without a deficit in social functioning). A trend towards lower spindle density was identified with increased clinical risk but this did not reach statistical significance (ANOVA, *F* = 2.31, *p* = .14; Supplementary Figure S3C). Further, spindle density did not correlate with the number of, or distress levels from, sub-clinical psychotic symptoms (number: *r* = −0.17, *p* = .262; distress: *r* = −0.21, *p* = .171). All power density and spindle analyses were calculated from a central C4 channel (Table 3), with similar findings observed for P4 and no differences identified for Fp2, F4, and O2 (see Supplementary Figure S3, Tables S2 and S3).

### HPA function

No differences were identified in the average cortisol awakening response (CAR) between high- and low-load exposure groups in terms of either delta changes across the recording period (average AUC_i_ across days; *W* = 215, *p* = .706), or the concentration of cortisol (nmol/L) at each time point (repeated-measures ANOVA, *p* > .05 in all cases).

### Subgroup differences—exploratory analysis

Based upon the identified high degree of variance in melatonin acrophase, significant differences in the High-load 2 from the High-load 1 subgroup were a later actigraphic sleep onset and a later subjective sleep mid-point. For subjective mental state, the High-load 2 subgroup showed the most extreme outcomes*—*the poorest subjective sleep quality, the greatest frequency of nightmares, the shortest EEG total sleep time, the lowest total number of spindles, and the greatest endorsement of psychotic-like experiences and number of risk factors for psychosis*—*when compared to the Low-load group, but this did not reach statistical significance against the High-load 1 subgroup (Table 4).

**Table 4.** Subgroup differences in melatonin metabolite, sleep and circadian parameters, and mental state measures

Parameter		Low load	High-load 1	High-load 2	Test value	*p*
Melatonin metabolite	Acrophase (h:min)	04:19 ± 1:09	03:50 ± 0:19	06:39 ± 1:13	21.85^#^	<.001^**LH**^
	Mesor (ng/h)	843.89 ± 247.88	946.99 ± 418.93	1135.93 ± 782.11	1.08^#^	.352
	Amplitude (ng/h)	940.36 ± 283.46	1069.51 ± 526.55	1309.84 ± 1038.54	1.03^#^	.370
Subjective Qs	Social jetlag (MCTQ, h:min)	1:00 ± 0:46	1:40 ± 1:01	1:11 ± 0:45	1.90”	.388
	Sleep mid-point (MCTQ, h:min)	04:33 ± 1:14	04:34 ± 0:44	06:02 ± 0:59	10.81”	.005^** LH**^
	Global sleep quality (PSQI)	3.38 ± 1.78	5.89 ± 2.30	7.64 ± 2.01	17.14”	<.001^ L^
	Nightmares (NFQ, per month)	0.56 ± 0.82	3.16 ± 3.57	3.36 ± 5.14	7.10”	.029^ L^
Actigraphy	Social jetlag (SJL, h:min)	0:43 ± 0:33	0:48 ± 0:53	0:25 ± 0:37	0.96^#^	.392
	Sleep mid-point (MSFsc, h:min)	04:44 ± 1:08	04:52 ± 1:19	05:41 ± 1:22	1.99^#^	.154
	Sleep onset (h:min)	00:17 ± 1:07	00:07 ± 0:37	01:19 ± 1:02	4.65^#^	.017^** LH**^
	Sleep offset (h:min)	08:34 ± 1:03	08:28 ± 1:03	09:27 ± 1:23	2.37^#^	.110
EEG	Total sleep time (h:min)	7:01 ± 0:45	7:15 ± 0:40	6:21 ± 1:03	3.18^#^	.055
	Sleep onset latency (min)	14.37 ± 10.21	13.32 ± 6.86	19.19 ± 14.93	0.81^#^	.455
	REM onset latency (min)	89.91 ± 30.45	92.81 ± 48.81	69.64 ± 29.45	1.41^#^	.260
	Sleep efficiency (%)	92.55 ± 2.82	93.60 ± 1.49	90.56 ± 5.41	1.77^#^	.188
	WASO (min)	19.24 ± 9.45	16.31 ± 6.10	20.21 ± 15.99	0.29^#^	.753
Spindle parameters	Spindle number (total sum)	1680 ± 228	1659 ± 198	1444 ± 361	2.70^#^	.082
	Spindle density (N/min)	6.73 ± 0.52	6.52 ± 0.51	6.52 ± 0.74	0.54^#^	.589
	Spindle frequency (Hz)	12.96 ± 0.23	13.15 ± 0.23	13.22 ± 0.33	3.24^#^	.052
	Sigma power (12–16 Hz; µV^2^)	3.02 ± 1.34	3.23 ± 1.78	2.71 ± 1.16	0.33^#^	.718
Mental state measures	PLEs (PQ-16)	1.06 ± 1.34	8.5 ± 2.33	9.09 ± 2.63	25.80”	<.001^ L^
	Risk factors (mean number)	2.13 ± 0.89	6 ± 1.51	7.18 ± 1.25	26.63”	<.001^ L^
	Depression (DASS-21)	3.63 ± 4.80	24.25 ± 7.52	21.45 ± 12.43	22.17”	<.001^L^
	Anxiety (DASS-21)	2.5 ± 2.58	17.75 ± 9.88	17.82 ± 9.40	24.44”	<.001^ L^
	Stress (DASS-21)	4.63 ± 4.72	23 ± 6.05	28 ± 9.34	25.47”	<.001^ L^
	Clinical psychosis risk (CAARMS)	0.00% [0]	75.00% [6]	81.81% [9]	20.01”	<.001^ L^

Shown are means± standard deviation or % [count]. Symbols denote different tests: ^#^ = analysis of variance, “ = Kruskal–Wallis test. ^L^ denotes a post hoc *t*-test significant difference for either High-load exposure subgroup to the Low-load exposure group. ^LH^ denotes a post hoc *t*-test significant difference for the High-load subgroup 2 to both the Low-load group and High-load subgroup 1. Low-load (*n* = 16), High-load 1 (*n* = 8), High-load 2 (*n* = 11).

## Discussion

In this study, we found that individuals with a high cumulative risk exposure for psychosis had a later circadian phase as assessed by at-home urinary melatonin profiles and reported subjective sleep disturbances compared to individuals with a low-risk exposure load. Objective polysomnographic sleep variables contributed little to explain subjective sleep disturbances. The multi-domain assessment of both subjective and objective circadian and sleep phenotypes used in this study, argues for a strong perceptual influence of negative affect on self-rated sleep quality and daytime function. Why might an individual with high environmental risk exposure show strong subjective but not objective sleep disturbances? Previously it has been shown that the PSQI does not correlate to either EEG or actigraphic measures and therefore subjective and objective assessments likely measure different aspects of sleep physiology [42–44]. Instead, the PSQI is thought to be a greater measure of psychology, which could reflect more negative cognitive viewpoints and general dissatisfaction [43]. In this sense, the higher PSQI scores in individuals with high cumulative risk exposures may reflect the observed elevated levels of anxiety, stress, and negative affect (depressive symptoms), producing an altered and more negative perception of sleep [45].

Despite this, one objective sleep difference was found. Although only marginal, the frequency of central and parietal sleep spindles was found to be higher in the High-load group. This property has not been reported to differ in individuals diagnosed with schizophrenia. However, in healthy individuals, when sleep pressure is low, for example following napping, spindle frequency increases with a dominance in centro-parietal (Cz Pz) brain location, and therefore this finding might point to a different sensitivity in sleep pressure [43].

More complex phenotypic patterns emerged from the circadian timing variability. Significantly later circadian timing was present in those with high cumulative risk exposures, demonstrated by the spread in melatonin peak times and sleep onset timing being significantly later relative to clock time in half of those with a high load of cumulative risk exposures. Individual sleep mid-points derived from chronotype assessments and habitual rest–activity phases matched the timing of their respective melatonin peak relatively closely, suggesting sleep processes take place in temporal coordination with the internal circadian phase. A similar circadian rhythm heterogeneity has been documented in individuals diagnosed with schizophrenia living in the community, whereby a subset of individuals had delayed and non-24-h melatonin rhythms [10]. These real-world, longitudinal data were recently combined with a predictive, biological-informed mathematical model [46], which revealed that sleep timing could be predicted from the ratio of daytime levels of light exposure against evening light levels.

Our results also suggest that if affective dysregulation is present, its effects extend to self-rating of sleep quality but not to self-rating chronotype, which corresponded well with physiological circadian timing. Late circadian chronotype, however, can be a consequence of innate physiology or habituation to factors interfering with typical bedtimes. This could include bright evening and late-night light exposure, perhaps as a result of dysfunctional thoughts relating to sleep. Our findings provide new insight for recognizing and differentiating between circadian, homeostatic, and affective processing contributions to self-reported sleep disturbances.

Finally, individuals with a high cumulative environmental risk exposure for psychosis reported more nightmares, although they were not more distressed by these nightmares. Nightmares are also more frequently reported in individuals with schizotypal personality [37], schizophrenia patients, and in UHR individuals [38–40] and are reported to be related to wake-time/daytime distress, emotional processing, and dissociative experiences [41]. These have collectively been linked to traumatic childhood experiences [42], which was one of the most prevalent risk factors in our group with high environmental risk exposures.

### Limitations

This study has many strengths through utilizing comprehensive phenotyping of developmental, behavioral, physiological, and biosocial measures providing an extended environmental risk grouping beyond clinical high-risk and genetically predisposed samples. However, certain limitations should be addressed. Despite taking great care in minimizing the probability of false-positive statistical outcomes, our findings would benefit from replication with a larger sample size and more non-student individuals. Measurement variability between methods (PSG, actigraphy) may be a result from only one night of PSG against 3 weeks of rest–activity monitoring, which highlights the importance of implementing longitudinal measurements to account for habitual sleep changes common in naturalistic settings. Furthermore, without longitudinal data, it cannot be determined what proportion of individuals will go on to develop a disorder, here specifically a psychotic disorder, and therefore what proportion represent relevant phenotypes for the refinement of environmental risk management and disease prevention. On the reverse of this, it is unknown how representative low-risk individuals were. The low amount of risk factors could represent an extreme group of individuals, although all were assessed as healthy, and there is no evidence to support a difference to the general population based on our previous survey data [8]. Notably, the Low-load group had one case of a family history of any mental disorder compared to 50% in the High-load group. While this might raise the question of genetic enrichment, a recent twin study addressed the interaction of heritability and environmental risk factors in young adults reporting that heritability actually became less important as environmental risk increased, referring to four dimensions of psychosis (paranoia, cognitive disorganization, grandiosity, and anhedonia), while hallucinations and “negative affect” were spared [47].

### Conclusion

Overall, this high-resolution phenotyping study indicates that individuals with a high load of risk exposure for psychosis comprise subgroups with heterogenous levels of physiological and psychological contribution to perceived poor sleep quality. The observed heterogeneity could, in part, be explained by a late circadian phase, but depressive vulnerability, level of life stress, and anxiety potentially combine to form dysphoric mood and cognitive bias toward a negative perception of sleep quality.

## Supplementary Material

zsac311_suppl_Supplementary_Material

## Data Availability

The data for this article will be shared upon reasonable request to the corresponding authors.

## Notes

The research for this study was performed at the Sleep and Circadian Neuroscience Institute (SCNi) at the University of Oxford.
