# Supplementary material for: Phenotypic divergence in sleep and circadian cycles linked by affective state and environmental risk related to psychosis
Source: Sleep. 2022 Dec 14;46(3):zsac311. doi: 10.1093/sleep/zsac311 (PMC9995776; doi:10.1093/sleep/zsac311)
Supplement: zsac311_suppl_Supplementary_Material [file zsac311_suppl_supplementary_material.docx]

**Phenotypic divergence in sleep and circadian cycles linked by affective state and environmental risk related to psychosis**

Purple, R.J.^1*^, Cosgrave, J.^2^, Alexander, I.^2^, Middleton, B.^3^, Foster, R.G.^2^, Porcheret, K.^4,5^, Wulff, K.^6,7*^

^1^ University of Bristol, School of Physiology Pharmacology and Neuroscience, Biomedical Sciences Building, University Walk, Bristol BS8 1TD, UK

^2^ University of Oxford, Sleep and Circadian Neuroscience Institute, Nuffield Department of Clinical Neurosciences, Sir William Dunn School of Pathology, South Parks Road, OX1 3RE, UK.

^3^ Chronobiology, Faculty of Health and Medical Sciences, University of Surrey, Guildford, UK

^4^ Norwegian Centre for Violence and Traumatic Stress Studies, University of Oslo, Norway

^5^Institute of Clinical Medicine, Faculty of Medicine, University of Oslo, Norway

^6^ Umeå University, Department of Radiation Sciences and Department of Molecular Biology, SE-901 87 Umeå, Sweden

^7^ Wallenberg Centre for Molecular Medicine (WCMM), Umeå University, SE-901 87 Umeå, Sweden

The research for this study was performed at the Sleep and Circadian Neuroscience Institute (SCNi) at the University of Oxford.

*Corresponding authors: ross.purple@bristol.ac.uk & katharina.wulff@umu.se

**Supplementary Information**

**Supplementary methods**

*Recruitment criteria*

The online survey used to recruit participants in this study was completed by the general population of Oxford, UK, and was freely accessible before and throughout the study period (from June 2014 – October 2015). Recruitment into the study began roughly 6 months after the survey became accessible. The majority of participants completed the online survey once within one month of being recruited into the study. The remaining participants were asked to re-do the survey to ensure they were still eligible.

The survey was comprised of an array of questions to assess risk factors for psychosis, mental health and sleep. Psychotic-like experiences, were assessed using the Prodromal Questionnaire-16 (PQ-16) with a score of more than 5 indicative of clinical risk for developing psychosis^1^. A Likert scale of distress levels for each item were also included. Risk factors were selected from a systematic literature search of systematic reviews and/or meta-analyses showing a clear, established positive association (regardless of strength) between the risk factor and a psychotic disorder. A total of 23 risk factors were assessed based upon previously used questionnaires where possible and binary scores for the presence or absence of a risk factor were calculated. Since psychotic disorders are associated with high comorbidity and overlapping symptoms to many other psychiatric disorders^2^, individuals were additionally assessed for symptoms of depression, anxiety and stress using the Depression Anxiety Stress Scale-21 (DASS-21)^3^. Individuals were given a binary score based on severity, with moderate or more (DASS-21 score of >9 for depression, >9 for anxiety or >18 for stress) having a score of 1. Help-seeking behaviour was also assessed to identify heightened distress levels with a score of 1 if individuals were seeking help for a psychotic-like experience from either counselling, therapy or a general practitioner. To select individuals, binary scores of risk factors for developing psychosis, symptoms of depression, anxiety, stress and help seeking behaviour were summed to produce a composite score. High-load participants were selected from the top 15% of the collective database sample (N=1898) for this composite score, in addition to having a positive screen on the PQ-16 (score of >5). High-load participants were thus selected for a high load of risk factors, sub-clinical psychotic experiences and general negative affect. A low-load group was selected from the bottom 15% of the composite score in addition to having a negative screen on the PQ-16 (<5). An outline of these recruitment characteristics are shown in Table S1 and Figure S1.

Table S1: Recruitment characteristics used to select high and low load groups (N=44).

| Recruitment characteristics | High-load | Low-load |
| --- | --- | --- |
| Psychotic-like experiences (PQ-16>5) | 22 (100%) | 0 (0%) |
| Depression (DASS-21>13) | 18 (82%) | 1 (5%) |
| Anxiety (DASS-21>9) | 19 (86%) | 2 (9%) |
| Stress (DASS-21>18) | 20 (91%) | 0 (0%) |
| Help seeking (yes) | 14 (64%) | 0 (0%) |
| Risk factors (mean number) | 6.8±1.5 | 2.1±0.9 |


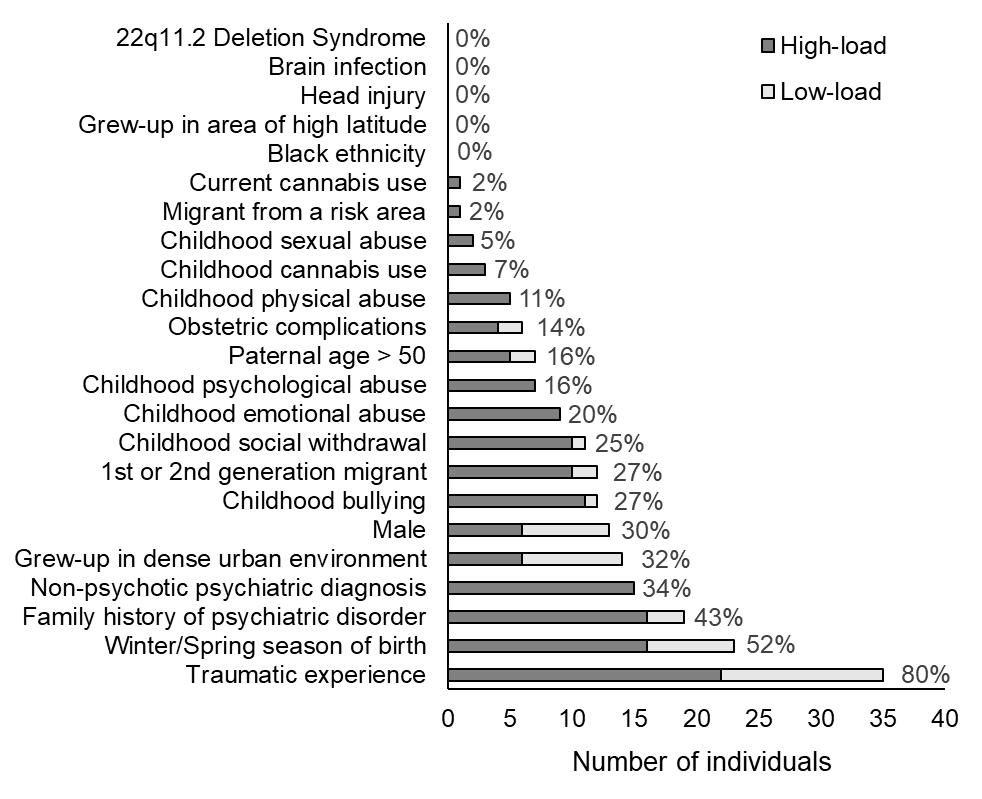


Figure S1: Prevalence of risk factors for developing psychosis between high and low load groups recruited into the study (n=22, n=22).

*Spindle analysis*

To detect spindles, the raw signal was filtered using an Infinite Impulse Response (IIR) bandpass Butterworth filter between 11-16Hz. Next, the instantaneous amplitude was computed using a Hilbert transform and an upper threshold was applied to detect spindle events, with a lower threshold used to determine the start and end of the spindle event. Visual scoring of sleep spindles, was carried out on 10% of the recordings (n=4) and compared with the automatic detection algorithm to determine the optimum thresholds. The upper threshold was set at the mean amplitude + 2 * standard deviation. The lower threshold was set at the mean amplitude + 1.5 * standard deviation. A maximal interruption interval of 300ms was allowed if the amplitude fell below the lower threshold and above again, ensuring a single spindle would not be detected as two. A duration threshold was also set limiting spindles to be detected between 0.5-2 seconds. Compared to visual detection, this algorithm produced a sensitivity of 78%, comparable to performance of many previously used algorithms^4^. Further, a previously published and validated algorithm^4^ was also applied to the visually scored sample producing a poorer sensitivity of 66%. Spindle characteristics were determined as follows: Amplitude, maximum amplitude within the spindle event; Duration, the difference between spindle start and end; Density, number of spindles per minute of NREM sleep; Frequency, number of amplitude peaks within the spindle event divided by the duration.

*Actigraphy*

For actigraphy, recordings were adjusted for daylight saving time by adding or removing an hour (based on whole day average) of data during the sleep period of the relevant night where applicable. If the watch had been removed for more than half an hour but less than three hours, the data was edited and replaced with average activity count for that day. Full days were excluded if the watch had been taken off for more than three consecutive hours. For the non-parametric circadian rhythm analysis, a minimum of two consecutive days were required and a weighted average was performed across each ‘block’ of days if not continuous. Actigraphic variables included: fragmentation index (degree of fragmentation of the sleep period – higher values indicated greater fragmentation), interdaily stability (degree of regularity in the activity-rest pattern scored between 0 to 1. A value of 0 indicates a complete lack of rhythm, a value of 1 indicates a perfectly stable rhythm), intradaily variability (the degree of fragmentation of activity-rest periods scored between 0 to 2. The higher score indicates higher fragmentation), L5 counts (average activity level for the least active 5 hours of the day), and M10 counts (average activity level for the most active 10 hours of the day).

*Melatonin*

Due to inaccuracies in participant data collection, certain manipulations were applied, where appropriate, to improve the fit of the cosinor model. If the model fit could not be improved (alpha<0.05), participants were excluded from the analysis. For four participants the first or last data point was removed before running through the cosinor model. Nine participants were excluded because of a non-significant cosinor output (alpha >0.05). Two further individuals also produced an alpha above 0.05 (0.064 and 0.075) but were included in the analysis since the aMT6s rhythm was highly congruent across the 48 hours, which was not seen in the excluded participants. A cut-off of alpha >0.10 is used in previous literature for this cosinor analysis and therefore our inclusion criteria was stricter than standard^5,6^. A total of 35 individuals were included in this analysis.

**Supplementary results**


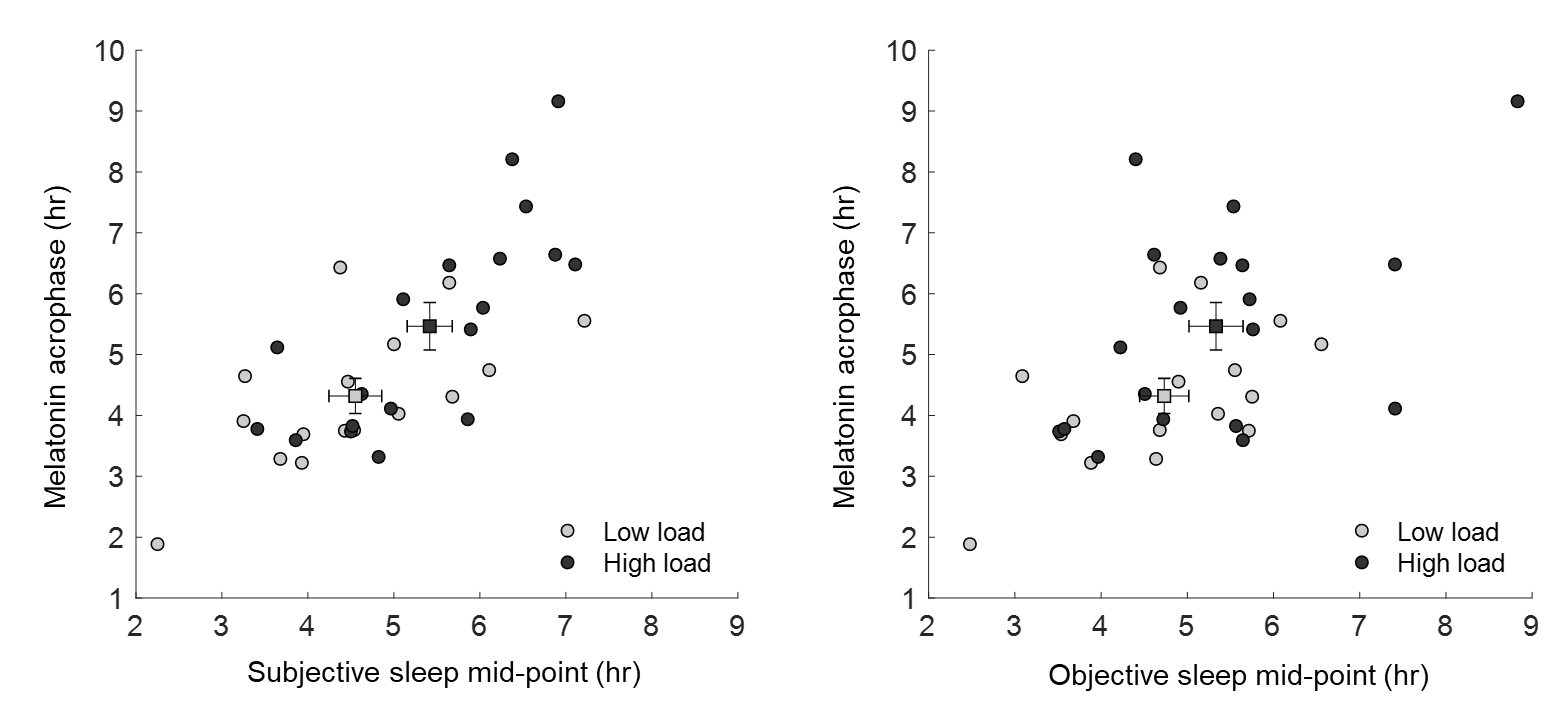


Figure S2: Scatterplots of melatonin peak in relation to subjective (Munich Chronotype Questionnaire, MCTQ) and objective (actigraphic) sleep mid-points. Squares denote mean with error bars denoting S.E.M.


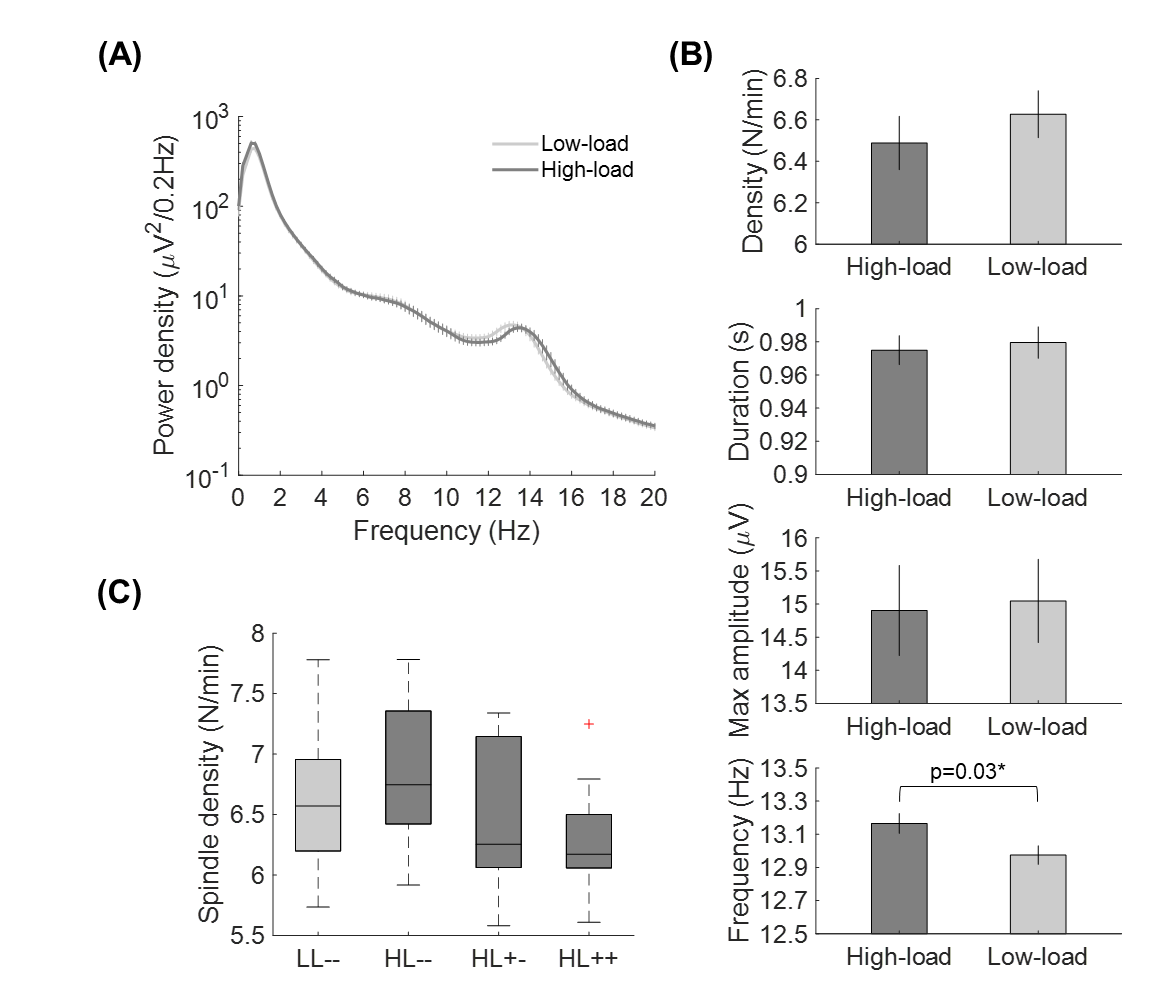


Figure S3: (A) Mean power density during artefact-free NREM sleep (0.2Hz resolution). (B) Average density, duration, amplitude and frequency of spindles during NREM sleep. Data is from central channel C4. Bars denote S.E.M. High-load, n=22; low-load, n=22. (C) Spindle density across different levels of clinical risk for developing psychosis (CAARMS Screen). LL=low-load group, HL= high-load group, -- = negative screen, +-= positive screen for symptoms of psychosis, but no deficit in social functioning, ++= positive screen for symptoms of psychosis and a deficit in social functioning. From left to right: n=22, n=5, n=8, n=9. Note y-axes truncated.

Table S2: Group differences in average spectral power density and spindle characteristics from central channel C4 during NREM sleep. High-load, n=22; low-load, n=22.

| Parameter | High-load | Low-load | t | p |
| --- | --- | --- | --- | --- |
| Power density |  |  |  |  |
| *Delta power (0.6-4Hz)* | 142.20±31.06 | 130.30±43.98 | 1.04 | 0.31 |
| *Theta power (4.2-8Hz)* | 10.96±3.34 | 10.92±4.12 | 0.04 | 0.97 |
| *Alpha power (8.2-12Hz)* | 4.34±2.38 | 4.50±2.36 | -0.21 | 0.83 |
| *Sigma power (12.2-16Hz)* | 2.94±1.35 | 2.94±1.19 | -0.00 | 1.00 |
| *Beta power (16.2-20Hz)* | 0.51±0.21 | 0.49±0.17 | 0.45 | 0.65 |
| Spindle characteristics |  |  |  |  |
| *Total number* | 1548±304 | 1551±295 | -0.03 | 0.98 |
| *Density (N/min)* | 6.49±0.61 | 6.63±0.53 | -0.81 | 0.42 |
| *Duration (s)* | 0.97±0.04 | 0.98±0.04 | -0.35 | 0.73 |
| *Amplitude (µV)* | 14.90±3.19 | 15.05±2.95 | -0.16 | 0.88 |
| *Frequency (Hz)* | 13.17±0.28 | 12.97±0.26 | 2.32 | 0.03* |

Table S3: Group differences in average spectral power density and spindle characteristics from frontal channel Fp2, F4, parietal channel P4 and occipital channel O2 during NREM sleep. High-load, n=22; low-load, n=22.

|  | Fp2 | | F4 | | P4 | | O2 | |
| --- | --- | --- | --- | --- | --- | --- | --- | --- |
| Parameter | t | p | t | p | t | p | t | p |
| Power density |  |  |  |  |  |  |  |  |
| *Delta power (0.6-4Hz)* | 1.75 | 0.088 | 1.45 | 0.156 | 0.66 | 0.512 | -0.16 | 0.874 |
| *Theta power (4.2-8Hz)* | -0.37 | 0.712 | 0.04 | 0.969 | -0.24 | 0.814 | -0.61 | 0.548 |
| *Alpha power (8.2-12Hz)* | -0.06 | 0.955 | -0.34 | 0.739 | -0.34 | 0.737 | -0.27 | 0.788 |
| *Sigma power (12.2-16Hz)* | 0.60 | 0.550 | -0.21 | 0.833 | -0.14 | 0.888 | -0.68 | 0.503 |
| *Beta power (16.2-20Hz)* | -0.12 | 0.902 | -0.51 | 0.613 | 0.27 | 0.789 | -0.29 | 0.776 |
| Spindle characteristics |  |  |  |  |  |  |  |  |
| *Density (N/min)* | -0.12 | 0.902 | -0.05 | 0.958 | -0.76 | 0.453 | -0.15 | 0.885 |
| *Duration (s)* | 1.04 | 0.305 | 0.26 | 0.797 | -0.26 | 0.793 | -0.22 | 0.826 |
| *Amplitude (µV)* | 0.56 | 0.576 | -0.05 | 0.958 | -0.25 | 0.801 | -0.68 | 0.498 |
| *Frequency (Hz)* | -0.18 | 0.857 | 0.57 | 0.574 | 2.08 | 0.043* | 1.09 | 0.283 |

*Melatonin cosinor curves*

Individual participant plots of midpoints between urine collection (/48hrs) and the corresponding melatonin amplitude (nh/hr). Plots show both raw data in red and the non-linear regression model fitted to the data in blue.

**Low-load group (n=16):**


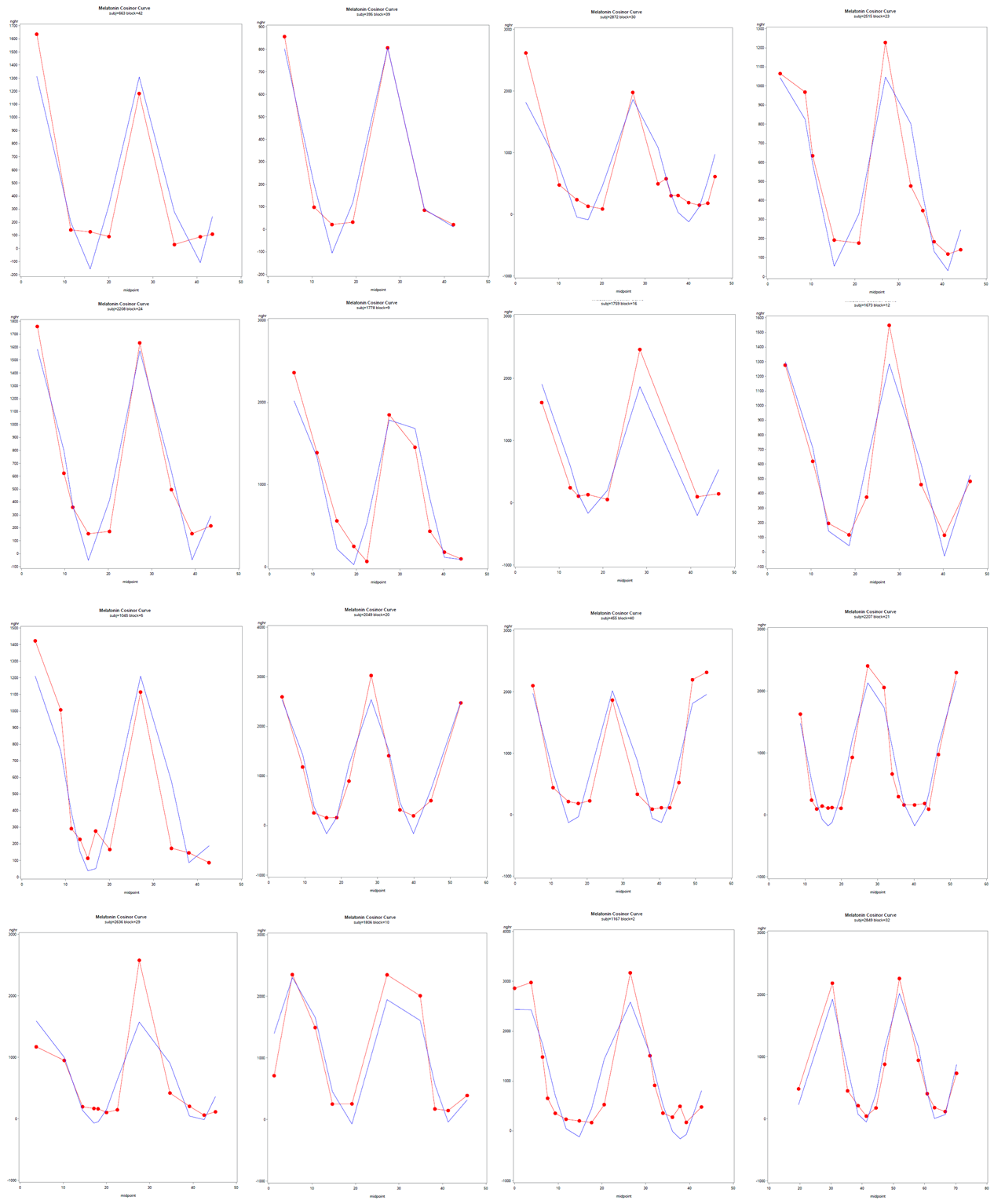


**High-load 1 (n=8):**

**
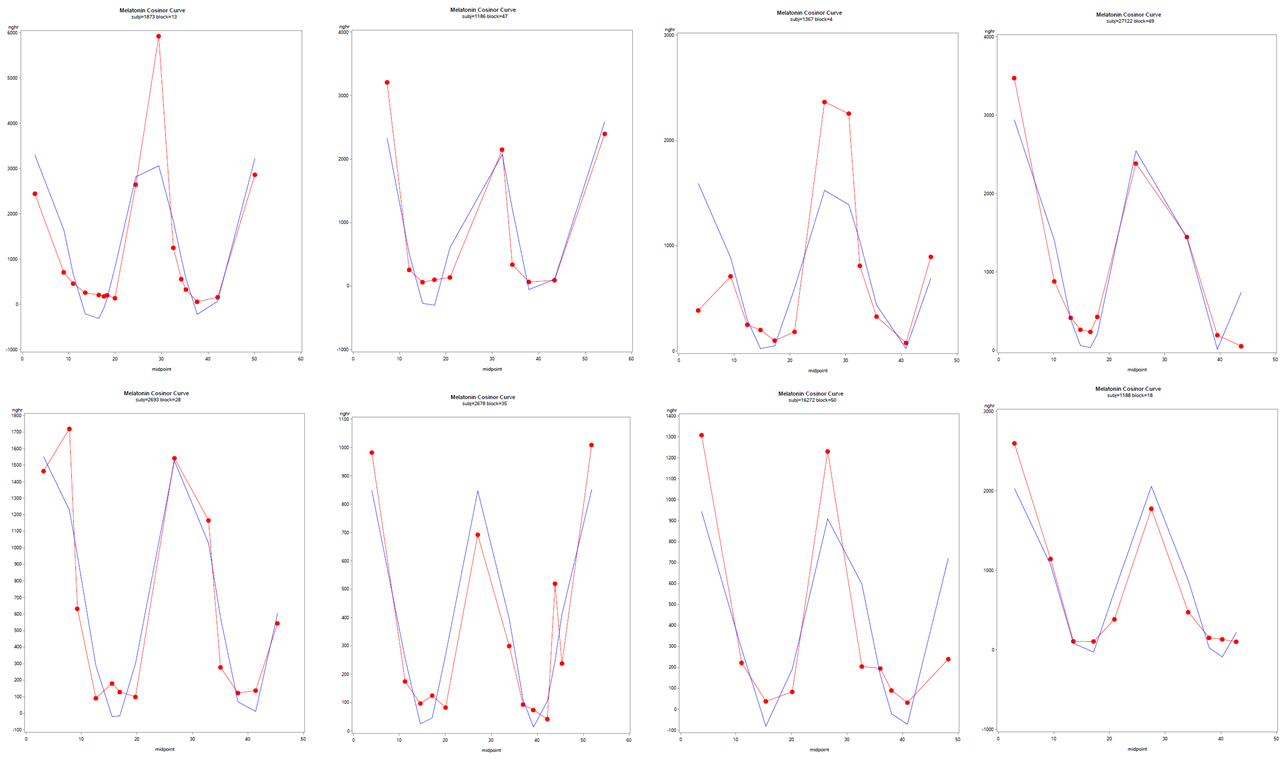
**

**High-load 2 (n=11):**

**
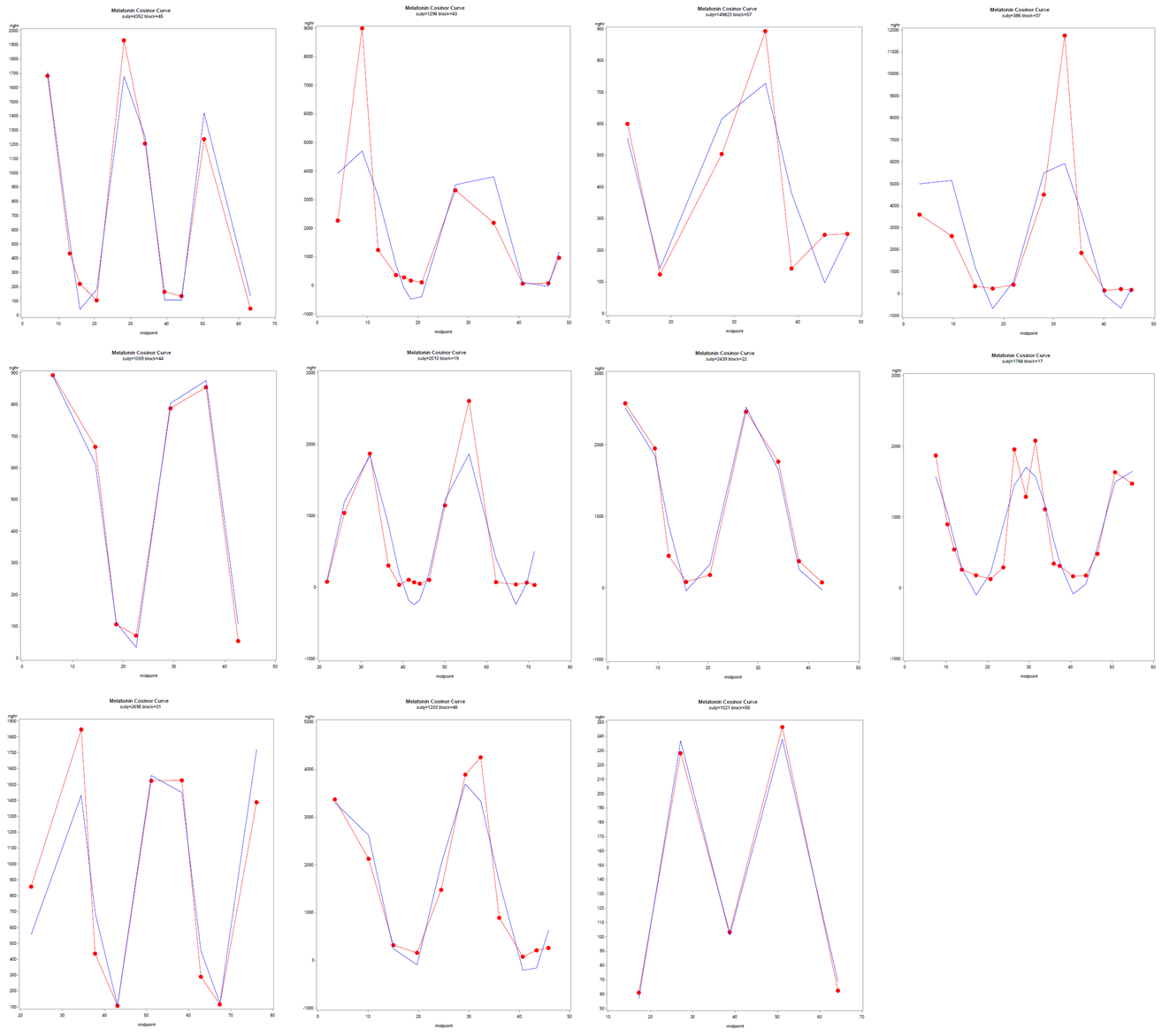
**

**References**

1. Ising HK, Veling W, Loewy RL, et al. The validity of the 16-item version of the Prodromal Questionnaire (PQ-16) to screen for ultra high risk of developing psychosis in the general help-seeking population. *Schizophr Bull*. 2012;38(6):1288-1296. doi:10.1093/schbul/sbs068.

2. Buckley PF, Miller BJ, Lehrer DS, Castle DJ. Psychiatric comorbidities and schizophrenia. *Schizophr Bull*. 2009;35(2):383-402. doi:10.1093/schbul/sbn135.

3. Henry JD, Crawford JR. The short-form version of the Depression Anxiety Stress Scales (DASS-21): construct validity and normative data in a large non-clinical sample. *Br J Clin Psychol*. 2005;44(Pt 2):227-239. doi:10.1348/014466505X29657.

4. Tsanas A, Clifford GD. Stage-independent, single lead EEG sleep spindle detection using the continuous wavelet transform and local weighted smoothing. *Front Hum Neurosci*. 2015;9:181. doi:10.3389/fnhum.2015.00181.

5. Ftouni S, Sletten TL, Nicholas CL, Kennaway DJ, Lockley SW, Rajaratnam SMW. Ocular Measures of Sleepiness Are Increased in Night Shift Workers Undergoing a Simulated Night Shift Near the Peak Time of the 6-Sulfatoxymelatonin Rhythm. *J Clin Sleep Med*. 2015;11(10):1131-1141. doi:10.5664/jcsm.5086.

6. Sletten TL, Ftouni S, Nicholas CL, et al. Randomised controlled trial of the efficacy of a blue-enriched light intervention to improve alertness and performance in night shift workers. *Occup Environ Med*. 2017;74(11):792-801. doi:10.1136/oemed-2016-103818.
